# RNA-NRD: a non-redundant RNA structural dataset for benchmarking and functional analysis

**DOI:** 10.1093/nargab/lqad040

**Published:** 2023-04-26

**Authors:** Nabila Shahnaz Khan, Md Mahfuzur Rahaman, Shahidul Islam, Shaojie Zhang

**Affiliations:** Department of Computer Science, University of Central Florida, Orlando, FL 32816, USA; Department of Computer Science, University of Central Florida, Orlando, FL 32816, USA; School of Computing and Design, California State University, Monterey Bay, Seaside, CA 93955, USA; Department of Computer Science, University of Central Florida, Orlando, FL 32816, USA

## Abstract

The significance of RNA functions and their role in evolution and disease control have remarkably increased the research scope in the field of RNA science. Though the availability of RNA structure data in PBD has been growing tremendously, maintaining their quality and integrity has become the greater challenge. Since the data available in PDB are results of different independent research, they might contain redundancy. As a result, there remains a possibility of data bias for both protein and RNA chains. Quite a few studies have been conducted to remove the redundancy of protein structures by introducing high-quality representatives. However, the amount of research done to remove the redundancy of RNA structures is still very low. To remove RNA chain redundancy in PDB, we have introduced RNA-NRD, a non-redundant dataset of RNA chains based on sequence and 3D structural similarity. We compared RNA-NRD with the existing non-redundant RNA structure dataset RS-RNA and showed that it has better-formed clusters of redundant RNA chains with lower average RMSD and higher average PSI, thus improving the overall quality of the dataset.

## INTRODUCTION

Ribonucleic acid (RNA) is a single-stranded macromolecule that not only carries genetic information but also performs different biological functions by forming various three-dimensional structures (1,2). Despite the rapid advancement in the field of biophysical RNA structure determination, researchers still face various challenges such as low-resolution data, missing coordinates, etc. These issues might cause variations in the determined RNA structures. As a result, multiple copies of a single RNA molecule with slight discrepancies are deposited on RNA 3D structure databases and this introduces the issue of redundancy. The structural comparison of RNA chains can be challenging and time consuming due to their structural complexity, which makes it harder to solve the issue of redundancy in RNA structure databases.

Research Collaboratory for Structural Bioinformatics Protein Data Bank (RCSB PDB) is an online public database for storing 3D crystal structures of biological macromolecules such as protein and nucleic acids (3). The PDB database is considered to be highly redundant as multiple depositions of the same molecular structure generated from different research works are accepted in PDB. In a paper published in 2009, Burra *et al.* anticipated that on average a protein structure is represented more than 4 times in PDB (4). For similar reasons, there exists a good amount of redundant structures of RNA macromolecules in this database. The source of this redundancy could either be their nucleotide sequences or their 3D structure or both. Moreover, the definition of redundancy could be different based on research perspective. Considering the necessity of organizing protein data and removing redundancy from protein databases, different non-redundant databases, hierarchical classification, and representative databases of proteins have been introduced. Some of these databases have been developed based on sequence similarity (5–7) while others concentrated on both sequence and structure (8–10).

With the advancement in the process of unraveling RNA structures, the number of RNA structures available in PDB is growing swiftly. Until 10 July 2021, PDB accommodated 5534 structures containing RNA chains. In order to explore RNA structural features and their relation to RNA function, analyzing RNA 3D structural data is imperative. But, due to the existence of high amount of redundancy, using the current version of PDB would introduce bias towards the characteristics of the same or similar molecular structures found more frequently in the database. Such data bias can significantly mislead the results of different evaluation algorithms and prediction models by reducing their performance and generalizability. Apart from that, the existence of huge amounts of redundancy in PDB causes unnecessary increase in computational time and resources. Also, for some macromolecules, incomplete and partial structures exist in PDB which might be less useful for future analysis and can be considered as data noise.

Finding the best possible representative for a redundant set of macromolecule structures will not only reduce this unnecessary data bias and noise, but it will also make further analysis of the dataset computationally more efficient. RNA 3D structure prediction (11,12), computational modeling and analysis (13), structural motif family and subfamily identification (14) are some of the major fields in which the redundant structures can affect significantly. Nevertheless, the work done so far for organizing and classifying RNA chains is mere compared to protein chains. Attempts have been made to structurally classify and annotate RNA chains in databases like SCOR, RNABase, etc. (15,16). Further works have been done to identify conserved RNA structures by classifying RNA chains based on their sequence, secondary structure, type of base pairing (17–22). In 2008, Abraham *et al.* (23) developed a database known as DARTS where they classified 1333 RNA structures into 94 clusters based on their spatial similarity.

In 2012, Ray *et al.* proposed a hierarchical database of RNA structures (HD-RNAS) where they classified RNA chains available at that time into nine different classes based on their functionality (24). Later, they classified the RNA chains further based on their organisms and generated a non-redundant version of the database by selecting the structures with the best resolution and R-factor. However, it depends on PDB provided molecular information to classify their function and does not consider the 3D structure similarity of RNA chains. The current version of this database only contains the classification of 2095 RNA chains and has not been updated since 2012. So, using HD-RNAS for further analysis of RNA chains is not a viable option anymore.

**Figure 1.**
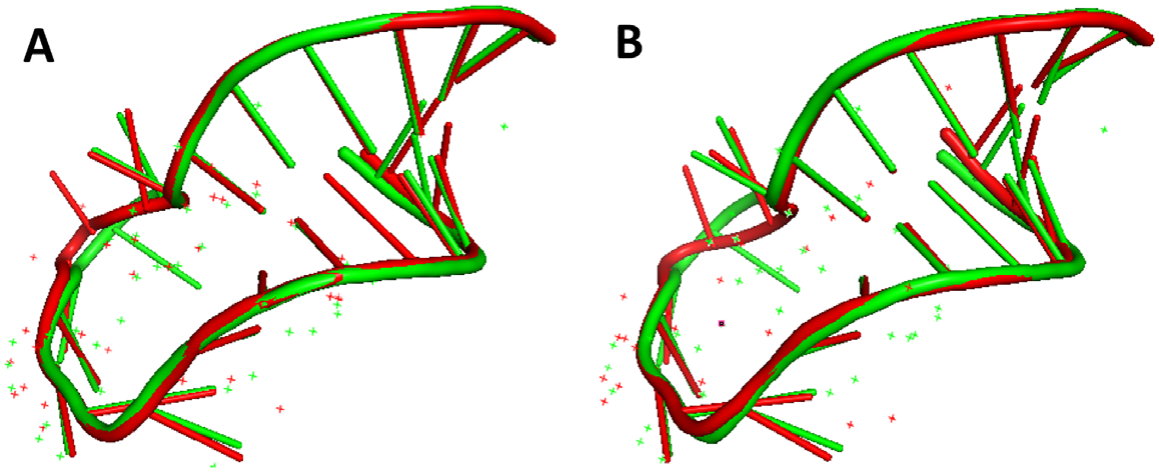
Structural alignment of RNA chains belonging to separate class in RS-RNA dataset. (**A**) Alignment of chain 6F4H_D (green) from class NR_all_64766.1 to chain 6F4H_B (red) from class NR_all_56099.1. (**B**) Alignment of chain 6F4H_D (green) and 6F4H_F (red) from class NR_all_64766.1 and NR_all_56099.1 respectively.

The most widely known non-redundant dataset of RNA 3D structures is the Representative Sets of RNA 3D Structures (RS-RNA) developed by Leontis *et al.* (25). Here, the authors clustered the redundant RNA structures together first by comparing their sequence and later through structural superposition and geometric analysis. Finally, they selected a representative with the highest number of base pairs per nucleotide for each redundant class. Though this non-redundant dataset is updated regularly, in the latest versions they use ‘Integrated Functional Element’ (IFE) instead of a single RNA chain as RNA molecule of interest. An IFE can consist of a single RNA chain or multiple RNA chains that have persistent RNA basepairing. This might create an issue where specifically non-redundant RNA chains are required for further analysis. Also, the current version of this dataset might contain some redundancy in the molecule level due to the presence of homologous IFEs. Apart from that, RS-RNA dataset still contain some cases of redundancy. For example, RNA chains B, F, D of PDB file 6F4H are redundant to each other as they have highly similar sequence and 3D structure (100% sequence identity with RMSD less than 1). But in version 3.176 of RS-RNA dataset, RNA chains B and F are assigned to class NR_all_56099.1 and RNA chain D is assigned to class NR_all_64766.1 while these three chains should be assigned to the same class. Figure 1 generated using tool PyMOL (26) shows how closely aligned the 3D structure of these chain pairs are. In RS-RNA, RNA chains are first divided based on a sequence identity threshold of 95%. This percentage might be too high as RNA chains can have significantly similar tertiary structure even with lower sequence identity. For example, RNA chain pairs 4LX6_A:4TZX_X and 4LX6_A:4TZY_X have RMSD respectively 0.51 and 0.58 with a sequence identity of 81.7%. Moreover, in RS-RNA, while considering structural similarity, they geometrically superposed only the aligned bases of the RNA chain sequences instead of aligning the whole RNA chain structures.

Keeping all of this in mind, we developed a non-redundant RNA structural dataset based on both RNA sequence and 3D structure coordinates. We refer to this dataset as RNA-NRD. Our main goal for developing this dataset is to identify redundant RNA chains based on both sequence and 3D structure similarity and represent a group of redundant RNA chains with a single representative chain. In RNA-NRD, two RNA chains belonging to the same source organism with a sequence identity ≥ 80%, RMSD value <4 Å and structural alignment ratio ≥80% are considered to be redundant to each other and are put together in the same cluster. Finally, the chain with the best quality score is selected as the representative of the cluster. We use the 3D structural alignment tool STAR3D which globally aligns the RNA chains. This improves the overall 3D structural comparison of RNA chains. To remove chain length dependency from RNA structural comparison, we use a parameter called structural alignment ratio which is the ratio of structurally aligned nucleotides with respect to the number of nucleotides with coordinates in the shorter RNA chain. In our dataset, we consider each RNA chain as a single entity. This not only handles redundancy among PDB files, but also handles redundancy within a PDB file. As depending on applications, the definition of redundant RNA structures can vary, we have generated another variation of RNA-NRD dataset where we don’t divide the RNA chains based on source organism. We refer to this dataset as RNA-NRD-without-Organism-Division. Both RNA-NRD and RNA-NRD-without-Organism-Division are updated on a regular basis and are publicly accessible to facilitate future research endeavors.

## MATERIALS AND METHODS

The whole process for generating the RNA-NRD dataset is illustrated step-by-step in this section. Also, the steps to generate RNA-NRD-without-Organism-Division and the pipeline for updating both the RNA-NRD and the RNA-NRD-without-Organism-Division datasets on a regular basis is discussed here in detail.

### Generating RNA-NRD dataset

**Figure 2.**
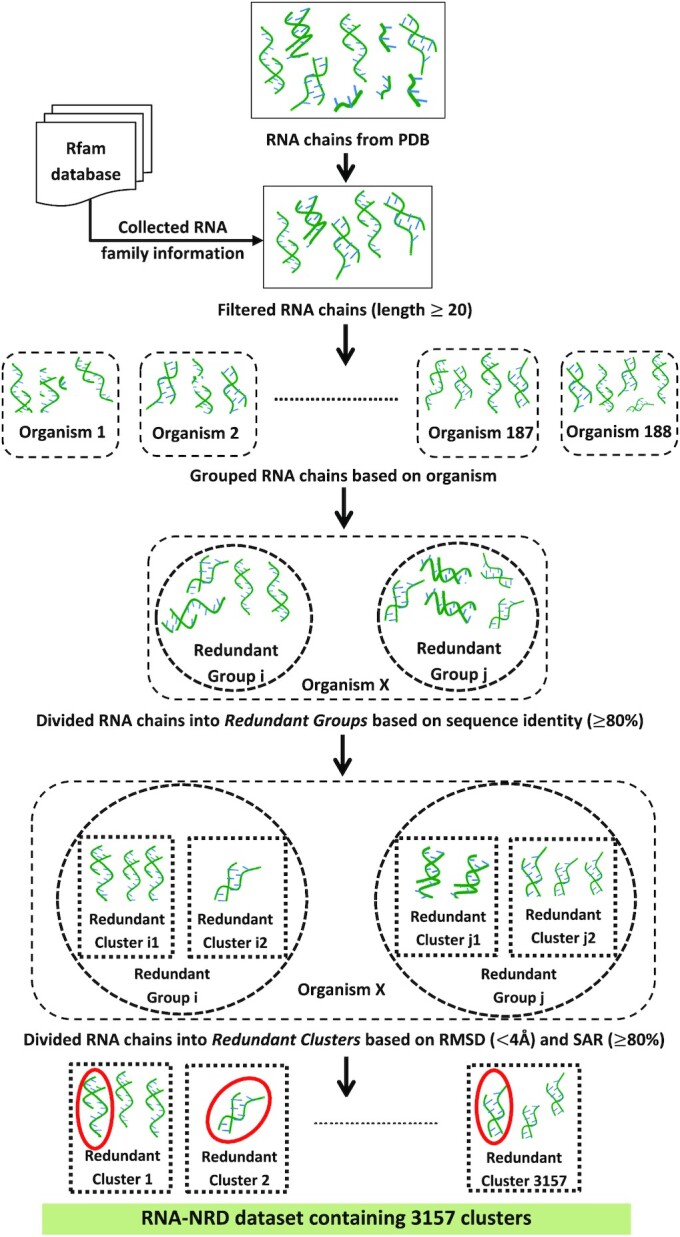
The pipeline for generating RNA-NRD dataset. Here, SAR represents structural alignment ratio. The solid rectangles show the set of input RNA chains and the dotted rounded rectangles show sets of RNA chains divided based on their organism. Redundant Groups are represented using dotted circles and Redundant Clusters are represented using dotted rectangles. Red ovals are used to highlight the representatives within a Redundant Cluster.

The different stages for generating the RNA-NRD dataset are shown in Figure 2. The steps are described below:

#### Collecting RNA chains

Nearly 12418 nucleic acid containing structures have been collected from PDB on 17 March 2021. From there, protein, DNA and NA-hybrid structures have been filtered out and a total of 9705 RNA chains having length ≥20 nucleotides are selected for further analysis. In PDB, each RNA chain contains a list of features. Among those, PDB ID, Experimental Method, Release Date, Resolution (Å), Sequence, Entity Polymer Type, Polymer Entity Sequence Length, Source Organism, Macromolecule Name and Chain ID, these 10 features are selected as they are more commonly used for RNA structure analysis.

#### Collecting Rfam RNA chain family

Rfam is a well-known database of RNA families where each family is represented by multiple sequence alignment and co-variance model (27–32). As of 17 September 2021, there were 4070 RNA families available on their website among which only 122 families were linked to the RNA 3D structures of PDB. We collected the family information of all such PDB IDs from the Rfam database and later integrated this family information into the RNA-NRD dataset. Rfam family names are included in the RNA-NRD dataset to give a better insight into the relation between RNA family and redundant RNA clusters. Such information can be helpful in future RNA structure analysis. Rfam collects RNA sequences from the European Nucleotide Archive (ENA) database (33) and not all RNA chain sequences present in PDB exists in the ENA database. Also, there exist some structure files in PDB whose RNA sequences contain invalid characters such as X and N. As a result, lots of PDB RNA chains don’t have a corresponding sequence in Rfam and so these chains haven’t been assigned to any of the RNA families in Rfam. For such RNA chains, their Rfam family name is assigned as ‘undefined’ in the RNA-NRD dataset.

#### Dividing RNA chains based on organism

Generally, PDB provides information regarding the source of RNA chains as organism name. In the RNA-NRD dataset, RNA chains belonging to different organisms are considered to be non-redundant. Hence, similarity checks between RNA chains are only conducted if they belong to the same organism. If the RNA chains are re-engineered RNA chains, then they are put together under the organism group ‘synthetic construct’. Including this, a total of 187 source organisms have been identified containing 8967 RNA chains. However, there are 738 RNA chains with no specified source organism. These RNA chains with missing source information are put together as a single organism under the name ‘others’, making a total of 188 organisms. A similarity check is performed within the chains belonging to this ‘others’ organism to see if there is any redundancy among them.

#### Analyzing sequence identity

In order to figure out possible redundancy between RNA chains, we first compare the sequence of the RNA chains and generate sequence identity. The sequence of two RNA chains are compared to each other only if the length of the longer RNA chain is not greater than twice the length of the shorter RNA chain. A pairwise comparison is done between all such pairs of RNA chains belonging to the same organism. For generating sequence alignment, Needleman-Wunsch sequence alignment algorithm (34) is used. Later, sequence identity is calculated by dividing the number of aligned nucleotides by the length of the shorter RNA chain. After analyzing the existing RNA chains, it was seen that structurally similar RNA chain pairs with RMSD < 4Å mostly tend to have a sequence similarity ≥80%. Hence, a sequence identity threshold of 80% has been considered to determine which RNA chains have a similar sequence. Considering higher threshold values such as 90–95% may segregate two RNA chains with a highly similar structure. As of today, no specific threshold of RNA sequence identity has been determined under which RNA 3D structures tend to vary significantly (35). Considering a high sequence identity threshold might introduce redundancy to the dataset as the sequences of two structurally similar homologous RNA can be different. On the other hand, considering a reasonably lower threshold for sequence identity significantly lowers the risk of introducing redundancy to the RNA-NRD dataset. Hence, all the RNA chains within a single organism having sequence identity ≥80% are assigned to the same group, defined as the ‘Redundant Group’.

#### Analyzing structural similarity

Comparing the structural similarity of RNA chains is of utter importance while removing redundancy. As per studies, homologous RNA molecules having similar functionality can be structurally very similar (36). RNA chains having high structural similarity but low sequence identity (<80%) are not considered redundant in RNA-NRD. To compare the structural similarity of two RNA chains within the same Redundant Group, we use two different approaches. If the RNA chains have 100% sequence identity, we use docking to generate the RMSD value. In this process, we superimpose the two structures using their sequence, calculate RMSD between each aligned pair of nucleotides and finally calculate the average RMSD. Otherwise, if the RNA chains have a sequence identity of <100%, we do a pairwise 3D structure comparison using the tool STAR3D (37). STAR3D is a global 3D structure alignment tool where the 3D conserved stacks are considered as anchors. Later, the loop regions within the matching stacks are compared which generates an overall RMSD value indicating the structural similarity between the two RNA chains. The reason behind using STAR3D is it works well for both homologous and non-homologous RNA chains and runs faster compared to other 3D structure alignment tools.

#### Generating clusters containing redundant RNA chains

**Figure 3.**
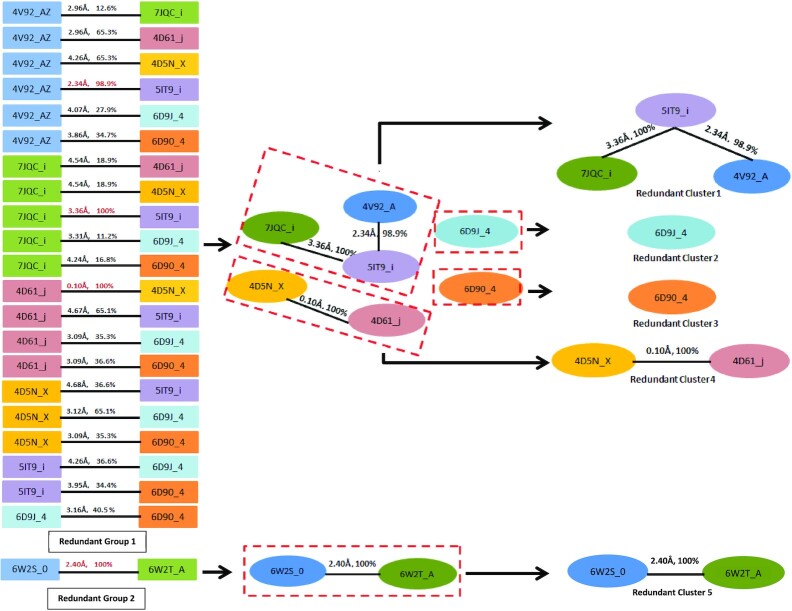
Step by step redundant RNA cluster generation process demonstrated using an example of organism *Cricket paralysis virus*. Organism *Cricket paralysis virus* contains nine RNA chains in total which are divided into two Redundant Groups (Redundant Group 1:4V92_AZ, 7JQC_i, 4D61_j, 4D5N_X, 5IT9_i, 6D9J_4, 6D90_4, and Redundant Group 2:6W2S_0, 6W2T_A) based on their sequence identity threshold. For both Redundant Group 1 and Redundant Group 2, all pairwise relations among the RNA chains are shown using black solid lines along with RMSD value and structural alignment ratio. Different RNA chains are represented using unique color codes. RMSD value <4 Å and alignment ratio ≥ 80% are highlighted using red color. Next, a graph is created keeping only the black solid lines with highlighted RMSD and structural alignment ratio. The connected components in the undirected graph are pointed out using red dotted lines. Each connected component of the graph forms a Redundant Cluster. Here, Redundant Group 1 forms four Redundant Clusters (Redundant Cluster 1, Redundant Cluster 2, Redundant Cluster 3, Redundant Cluster 4) whereas Redundant Group 2 forms a single Redundant Cluster (Redundant Cluster 5).

After performing pairwise structural alignment within a Redundant Group, all the RNA chains having RMSD value <4 Å and structural alignment ratio ≥80% are assigned together to a single ‘Redundant Cluster’. This is done by generating an undirected graph where the nodes represent the RNA chains and edges are drawn between two chains if their RMSD value <4 Å and structural alignment ratio ≥80%. The structural alignment ratio between two RNA chains is calculated by dividing the number of structurally aligned nucleotides with the number of nucleotides of the shorter RNA chain that have corresponding 3D coordinates in the PDB file. Here, two superimposed nucleotides are considered to be structurally aligned if their average spatial distance is <4 Å. Structural alignment ratio is used to remove the length dependency of RNA chain structural comparison. Each connected component in the undirected graph represents a Redundant Cluster. We use the concept of transitivity, meaning if two chains are redundant by definition, the other RNA chains redundant to those two chains will also be considered redundant to each other and will belong in the same Redundant Cluster. For better understanding, this whole process is demonstrated in Figure 3 where the organism *Cricket paralysis virus* is used as an example. This organism contains 9 RNA chains in total. First, these chains are divided into two Redundant Groups based on their sequence identity. RNA chains having sequence identity ≥ 80% are assigned to the same Redundant Group. Later, based on structural similarity, ‘Redundant Group 1’ is divided into four Redundant Clusters, and ‘Redundant Group 2’ forms only one Redundant Cluster. All the RNA chains belonging to the same Redundant Cluster are considered to be redundant. In total, a number of 3157 Redundant Clusters are generated in the RNA-NRD dataset.

#### Selecting representative

For each Redundant Cluster, the RNA chain with the best quality score is selected as the representative. The quality score of each RNA chain is calculated based on the number of nucleotides that have corresponding coordinates (*n*), number of base pairs (*b*), structure resolution (*r*) and chain degree (*d*). For each chain, the values *n* and *r* are collected from the corresponding PDB files. The value *b* is generated using the tool DSSR (38). Value *d* represents the number of edges each chain has with other chains in the connected graph generated based on RMSD value and structural alignment ratio. Here, equation 1 uses the weighted sum method to calculate the quality score. The variables *r*_0_, *n*_0_, *b*_0_ and *d*_0_ represent normalized *r*, *n*, *b* and *d* respectively. Additionally, variables *w*_1_, *w*_2_, *w*_3_ and *w*_4_ represent the weights assigned to each of these variables. For a RNA chain in a certain cluster, the values *n*, *b* and, *d* are normalized by dividing them with their maximum value within that cluster. Since lower value of *r* represents higher resolution, *r*_0_ is calculated by first dividing *r* with the maximum resolution and then subtracting the result from 1.


(1) $$\begin{equation*} Quality\ Score = r_0 * w_1 + n_0 * w_2 + b_0 * w_3 + d_0 * w_4\ \ \ \end{equation*}$$

Here, higher weight 0.4 (*w*_1_) is assigned to resolution (*r*) as RNA chains with higher resolution are considered to be more accurate in general. Also, RNA chain structures containing more nucleotides (*n*) and base pairs (*b*) are more likely to be better representatives and they are both assigned a weight of 0.25 (*w*_2_ and *w*_3_). Apart from that, chain degree (*d*) is also considered to prioritize RNA chains that are strongly connected in a cluster as they are structurally similar to most of the RNA chains belonging to that cluster and hence are assigned a weight of 0.1 (*w*_4_). The total of all the weights assigned to these four variables is 1. The quality score calculated for each RNA chain ranges between 0 and 1. In case multiple RNA chains have the same quality score in a single Redundant Cluster, then the representative will be selected based on other criteria in the following order of priority: (i) experimental method, (ii) chain length and (iii) release date. Among different experimental methods, X-ray diffraction is considered to have higher quality (39,40) and is preferred over other methods. Also, RNA chains with the latest release dates are more up-to-date, and longer RNA chains make better candidates as representatives as they cover more structural features.

### Generating RNA-NRD-without-Organism-Division dataset

To generate this version of the dataset, we compared the representatives of each Redundant Cluster in the RNA-NRD dataset. The goal is to merge redundant RNA chains from separate organisms and assign them in the same cluster. If two representatives from two different clusters have sequence identity ≥80%, RMSD <4 Å and structural alignment ratio ≥80%, then all the RNA chains belonging to those two clusters are merged together. Next, a pair-wise sequence and structural comparison is done among all these merged RNA chains and an undirected graph is generated by connecting the RNA chains which have both sequence and structural similarity to make sure that no non-redundant RNA chains are assigned together in the same cluster. Finally, following the concept of transitivity, each connected component of that graph is generated as a new cluster for redundant RNA chains where the RNA chains do not necessarily come from the same source organism. For each newly generated cluster, a representative with the highest quality score is selected. Here, quality score is calculated using the same procedure as described for RNA-NRD dataset in the ‘Selecting representative’ section.

### Pipeline for updating RNA-NRD dataset

**Figure 4.**
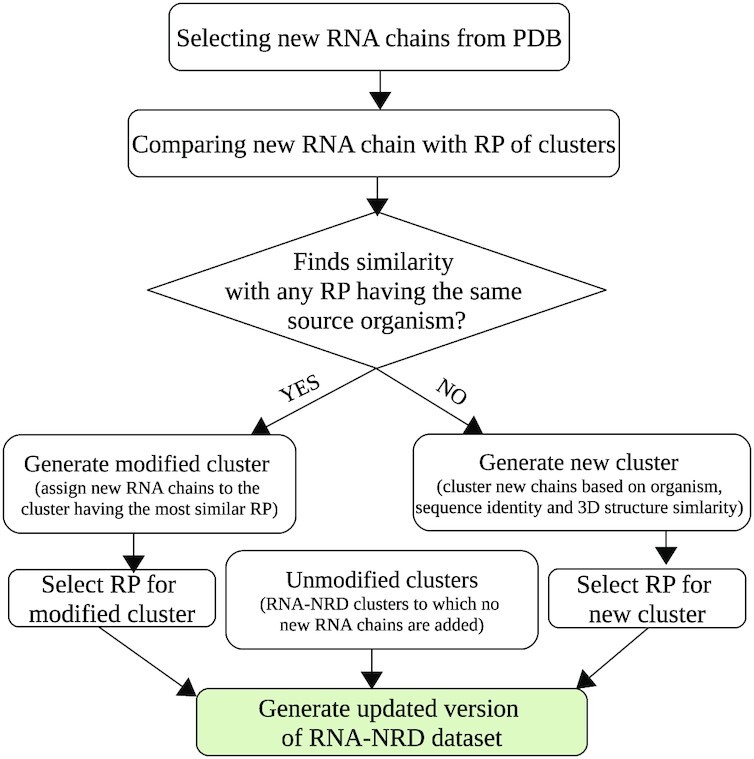
The pipeline for updating the RNA-NRD dataset. Here, ‘RP’ represents cluster representative.

PDB is a highly growing database. The PDB statistics (https://www.rcsb.org/stats/growth/growth-rna) of the last 10 years shows that on average, it releases 71 new RNA-only structures per year. To incorporate the newly introduced RNA chains in the non-redundant dataset, regularly updating the RNA-NRD dataset is mandatory. Hence, a fast and optimized pipeline has been developed to update the RNA-NRD dataset every 3 months. Instead of doing a pairwise comparison within all RNA chains of a certain organism, this pipeline will only compare the newly added RNA chains of PDB to the representative RNA chains (RP) with the same organism introduced in the previous version of the RNA-NRD dataset. Thus, it will generate a new version that includes the new PDB RNA chains. Figure 4 highlights the overall steps required in the RNA-NRD update pipeline. These steps have been discussed briefly in this section. With slight adjustments, this pipeline can also be used to update the RNA-NRD-without-Organism-Division dataset on a regular basis. To maintain consistency and avoid discrepancies in the dataset generated using the ‘original pipeline’ (shown in Figure 2) and the ‘update pipeline’ (shown in Figure 4), we plan to generate a non-redundant dataset using the original pipeline once a year.

#### Selecting new RNA chains

The newly added RNA chains in PDB are collected along with their important features. Other necessary information such as Rfam family name, number of base pairs, number of residues with coordinate in PDB file is also generated for these new RNA chains. New RNA chains having sequence length <20 and missing residue coordinate percentage >95% are filtered out and the rest are considered for further analysis.

#### Checking for similarity against representatives

The new RNA chains are compared against the cluster representatives (RPs) of the last version of the RNA-NRD dataset. For a certain new RNA chain, RPs having the same source organism, sequence identity ≥80%, RMSD <4 Å and structural alignment ratio ≥80% are considered to be similar. Here, the structural alignment ratio is calculated by dividing the number of structurally aligned nucleotides by the number of residues with coordinate of the new RNA chain instead of the number of residues with coordinate of the smaller RNA chain as the main focus of this comparison is the new RNA chain. While updating the RNA-NRD-without-Organism-Division dataset, the only difference is that the new RNA chains are compared against all the RPs regardless of their source organism.

#### Generating modified clusters

For a new RNA chain, among all the similar RPs, the RP having the maximum similarity is considered to be a match. As the new RNA chain is significantly similar to this RP, so it can be considered redundant to this RP and its cluster members. Thus, the new RNA chain is assigned to the cluster of that matched RP. Later, for such modified clusters, a new representative is selected based on the quality score. The quality score is generated using Equation (1).

#### Generating unmodified clusters

If the RP of a RNA-NRD cluster does not match with any new RNA chain, then no new RNA chains will be added to that cluster and it will remain unmodified. While generating the updated version of RNA-NRD dataset, these unmodified clusters will be added just as it is to the updated version.

#### Generating new clusters

New RNA chains, which are not similar to any of the current cluster representatives, are compared among themselves. For that, a similar pipeline is used as shown in Figure 2. First, the RNA chains are divided based on their source organism. Next, the chains are divided into separate groups based on sequence identity where chains having the same organism and sequence identity ≥80% are assigned to the same group. After that, their structural similarity is compared using the RMSD value and structural alignment ratio. Chain pairs having RMSD <4 Å and structural alignment ratio ≥80% are assigned in the same cluster and are considered to be redundant to each other. Finally, the RNA chain with the maximum quality score is nominated as the representative of that new cluster. The only difference for updating the RNA-NRD-without-Organism-Division dataset is that, the RNA chains are not divided based on their source organism and all the new RNA chains which are not similar to any of the current cluster representatives, are compared among themselves to check for similarity.

## RESULTS

### RNA-NRD dataset

RNA-NRD is a dataset that contains the redundant RNA chains together in a cluster and presents a representative RNA chain for each cluster. The first version of the RNA-NRD dataset is shown in Supplementary Table S3. It consists of a total of 3157 clusters containing 9705 RNA chains. In RNA-NRD, each redundant cluster also contains other relevant information such as source organism, macromolecule name collected from PDB, and family name collected from Rfam. RNA-NRD only contains RNA chains having a length ≥20 nucleotides as RNA chains shorter than that are considered less significant. The description of the present attributes of RNA-NRD is shown in Table 1.

**Table 1.** RNA-NRD dataset attribute list

Attribute no.	Attribute name	Description
1	Cluster ID	Unique identifiers assigned to Redundant Clusters
2	Representative	Representative RNA chain for each redundant cluster
3	Redundant Cluster	Cluster containing redundant RNA chains
4	Organism	PDB assigned source organism to RNA chains
5	Macromolecule Name	PDB assigned macromolecule names to RNA chains
6	Rfam Family Name	Rfam assigned RNA family name to RNA chains

### Benchmarking RNA-NRD dataset

In order to benchmark the RNA-NRD dataset, we compared it to the most widely used RNA non-redundant structural dataset known as ‘Representative Sets of RNA 3D Structures’ (RS-RNA) (25). For comparing these two datasets, we used two separate 3D structural alignment tools STAR3D and RNA-align. Parameters such as Average (Avg) Cluster RMSD, Average PSI (percentage of structural identity), and Average TM-score have been used to give an idea about how structurally similar the RNA chains within each redundant cluster are in both of the datasets. Here, PSI represents the percent ratio of structurally aligned nucleotides of two RNA chains with respect to the number of nucleotides of the shorter RNA. While making the comparison between the RNA-NRD and the RS-RNA datasets, only the common RNA chains between the two datasets are considered. First, an overall comparison is shown between the two datasets in Table 2. Then a more elaborate comparison is shown for a selected set of organisms in Table 3. Finally, an application based comparison is shown in Tables 4 and 5 where it is seen that motif subfamily generator tool RNAMotifContrast performs better using RNA-NRD dataset compared to the RS-RNA dataset.

**Table 2.** Overall comparison of RS-RNA and RNA-NRD dataset

					Overall similarity			
Dataset	#Chains	#Clusters	Avg RMSD (Å)	Avg PSI	#Common chains	#Similar clusters	#Common RPs	Similarity (%)
RS-RNA	13376	3059	2.29	0.87	9019	919	981	95.74%
RNA-NRD	9705	3157	**1.36**	**0.91**				

The better Avg RMSD and Avg PSI values are set to bold.

**Table 3.** Comparison of RNA chain clusters within RS-RNA and RNA-NRD dataset for the selected nine organisms

					#Clusters having		Total		RNA-align avg. RMSD(Å)/TM-score	
Organism	#Chains	#Total clusters	#Single-chain clusters	Cluster ratio	RMSD +/**-**/=	PSI **+**/-/=	RMSD +/**-**	PSI **+**/-	RNA-NRD	RS-RNA
*Deinococcus radiodurans*	70	3	0	23.3	0/0/3	0/0/3	*0/0*	*0/0*	*1.10/0.95*	*1.10/0.95*
*Escherichia virus*	61	7	6	8.7	0/0/1	0/0/1	*0/0*	*0/0*	*0.78/0.58*	*0.78/0.58*
*Haloarcula marismortui*	163	15	7	10.9	1/2/5	1/2/5	0.006/**0.01**	0.007/0.11	1.03/0.76	0.90/0.79
*Oceanobacillus iheyensis*	38	6	2	6.3	0/3/1	2/1/1	0/**2.39**	**0.05**/0.04	**1.25**/**0.93**	1.30/0.90
*Oryctolagus cuniculus*	303	45	29	6.7	5/4/7	4/4/8	2.34/**11.07**	**0.82**/0.06	**1.73**/**0.95**	2.92/0.67
*Roseobacter sp*	40	3	1	13.3	0/0/2	0/0/2	*0/0*	*0/0*	*1.22/0.23*	*1.22/0.23*
*Staphylococcus*	83	9	2	9.2	3/0/4	0/2/5	2.33/0	0/0.13	**1.60**/0.94	1.65/0.95
*Thermotoga*	31	5	1	6.2	0/3/1	1/1/2	0/**0.99**	**0.1**/0.006	**0.88**/**0.90**	0.94/0.73
Unidentified	72	9	6	8	0/2/1	2/0/1	0/**3.49**	**0.07**/0	**0.001**/**1.0**	0.04/0.94

Better performance by RNA-NRD is set to bold and equal performance by both dataset are set to italic. First column shows organism name and next four columns respectively show total number of chains, number of clusters, number of clusters containing single chain, and cluster ratio for each organism in RNA-NRD dataset. Sixth column shows the number of clusters having positive, negative, and zero RMSD-diff separated by ‘/’. Seventh column shows number of clusters having positive, negative, and zero PSI-diff separated by ‘/’. Eighth column shows the total positive and negative RMSD-diff of all the clusters in an organism separated by ‘/’. Similarly, ninth column shows the total positive and negative PSI-diff separated by ‘/’. Tenth and eleventh columns show RNA-align tool generated average RMSD and TM-score of all the clusters belonging to a specific organism separated by ‘/’.

**Table 4.** Comparison of RS-RNA and RNA-NRD dataset based on motif subfamilies generated using RNAMotifContrast tool

	RS-RNA				RNA-NRD			
Family name	No. of subfamilies	No. of redundant subfamilies	No. of total RNA chain pairs with similar organism	No. of redundant RNA chain pairs with similar organism	No. of subfamilies	No. of redundant subfamilies	No. of total RNA chain pairs with similar organism	No. of redundant RNA chain pairs with similar organism
*C-loop*	6	1	5	1	6	2	5	2
*E-loop*	3	1	9	2	3	1	6	1
*Hook-turn*	3	0	1	0	3	0	1	0
*Kink-turn*	4	1	20	5	6	1	12	3
*L1-complex*	2	1	1	1	2	1	1	1
*Rope-sling*	1	0	3	0	2	0	1	0
*Sarcin-ricin*	4	1	22	1	6	0	24	0
*T-loop*	1	0	0	0	1	0	0	0
*Tandem-shear*	3	1	23	1	2	0	20	0
*Tetraloop-receptor*	2	0	22	2	2	0	17	0
*Reverse Kink-turn*	3	0	0	0	3	0	0	0
*Total*	32	6	106	13	36	5	87	7

**Table 5.** Overall comparison of redundancy in RNA motif subfamilies generated by RNAMotifContrast using RS-RNA and RNA-NRD dataset

Percentage criteria	RS-RNA	RNA-NRD
Percentage of non-redundant families	45.45%	**63.64%**
Percentage of non-redundant subfamilies	81.25%	**86.11%**
Percentage of non-redundant RNA chain pairs	87.74%	**91.95%**
(with similar organism)		

The best performance is set to bold.

#### Comparison of RNA-NRD with RS-RNA

The overall comparison between the RNA-NRD and the RS-RNA datasets is shown in Table 2. Version 3.176 containing RNA chains of all resolutions of RS-RNA dataset has been used here for comparison. In total, this version contained 13376 RNA chains and RNA-NRD first version contains 9705 RNA chains. This number is significantly less in RNA-NRD because it discards all RNA chains having sequence length <20. The number of common RNA chains between the two datasets is 9019 and only these chains have been considered for comparison. RS-RNA and RNA-NRD divided the RNA chains into 3059 and 3157 final clusters respectively. Among them, there are a total of 919 similar clusters which contain the same RNA chain members. Apart from that, 981 of the clusters share a common representative and the two datasets have an overall similarity of 95.74%. This means 95.74% of the 9019 common RNA chains in RNA-NRD belong to a compatible cluster in RS-RNA. In order to determine compatibility, each cluster of the RNA-NRD dataset is compared to all the clusters of the RS-RNA dataset. A cluster in the RNA-NRD dataset is considered compatible with a cluster in the RS-RNA dataset when they share the maximum number of common RNA chains compared to the other existing clusters in the RS-RNA dataset. For example, suppose, Cluster A from RNA-NRD is compatible with Cluster B in RS-RNA. This means, after comparing Cluster A to all the clusters of RS-RNA dataset, it is seen that Cluster A shares maximum number of common RNA chains with Cluster B. Average RMSD and PSI values are calculated for both datasets by generating the RMSD and PSI values between all the RNA chain pairs within each cluster. To generate RMSD value and number of aligned nucleotides between two RNA chains, STAR3D is used. For both of the datasets, first the average RMSD and PSI values were generated for each cluster and then, their summation was divided by the number of total clusters. The overall average RMSD and PSI values are 2.29 and 0.87 for RS-RNA while for RNA-NRD, the average RMSD and PSI values are 1.36 and 0.91. Quite evidently, RNA-NRD has a lower average RMSD and higher average PSI value which shows that its clusters are better formed and the RNA chains within its clusters tend to be structurally more similar. In a redundant cluster, it is important to have structurally similar RNA chains as these chains are considered redundant to each other and only the representatives of such chains are taken into consideration.

#### Organism specific comparison of RNA-NRD with RS-RNA

To give a better idea about how the clusters in RNA-NRD have better structural similarity compared to the compatible clusters proposed in RS-RNA, we showed the comparison of the clusters for some selected well-known organisms in Table 3. From RNA-NRD, 12 specific organisms are selected based on the number of RNA chains they contain and their Cluster Ratio. For each organism, Cluster Ratio is calculated by dividing the number of RNA chains in that organism by the total number of clusters present in that organism. Organisms having large number of RNA chains are better candidates for quality assessment. Cluster Ratio is taken into consideration to filter out organisms having too many single RNA chain clusters. Having too many single RNA chain clusters within an organism will affect the cluster quality assessment as no structural similarity assessment can be performed for such clusters.

Among all the 188 organisms, 12 organisms having RNA chains ≥30 and cluster ratio ≥5.5 have been finally selected for the comparison. However, among these 12 organisms, *Escherichia coli*, *Saccharomyces cerevisiae* and *Thermus thermophilus* contain too many RNA chains. Hence, generating their RMSD and TM-score using the structural alignment tool RNA-align (41) is computationally very expensive. So, the comparison of the remaining nine organisms is finally shown in Table 3. Here, the structural alignment tool RNA-align is used to show a non-biased quality assessment comparison between RNA-NRD and RS-RNA datasets. In order to compare the structural similarity of clusters between the two datasets, first the average RMSD and average PSI of each cluster for both datasets are calculated using STAR3D. Then RMSD-diff and PSI-diff are calculated for all the clusters present in the RNA-NRD dataset.

RMSD-diff is the difference between the average RMSD values of two compatible clusters where the first cluster belongs to RNA-NRD and the second one belongs to RS-RNA. Similarly, PSI-diff shows the difference between average PSI values of two compatible clusters of RNA-NRD and RS-RNA. Suppose, c_*i*_ is a cluster in the RNA-NRD dataset and c_*j*_ is its compatible cluster from the RS-RNA dataset. Then, the RMSD-diff of cluster c_*i*_ of the RNA-NRD dataset has been calculated by subtracting the average RMSD of c_*j*_ from the average RMSD of c_*i*_ cluster. If c_*i*_ cluster’s average RMSD is less than c_*j*_ cluster’s average RMSD, then the RMSD-diff value will be negative. This means c_*i*_ cluster of the RNA-NRD dataset is structurally more similar than its compatible cluster c_*j*_. On the other hand, a positive RMSD-diff value will mean c_*j*_ cluster of the RS-RNA dataset is structurally more similar than c_*i*_. In case both of the clusters have equal average RMSD values, then RMSD-diff will be 0, and such cases have been represented using the ‘=’ symbol. If any of these two clusters contain a single RNA chain, then RMSD-diff will be ‘Null’. Similarly, PSI-diff has been calculated for each cluster in the RNA-NRD dataset. But in case of PSI, having a positive PSI-diff value will mean c_*i*_ cluster of the RNA-NRD dataset is structurally more similar than its compatible cluster c_*j*_ and a negative PSI-diff value will mean the opposite.

**Figure 5.**
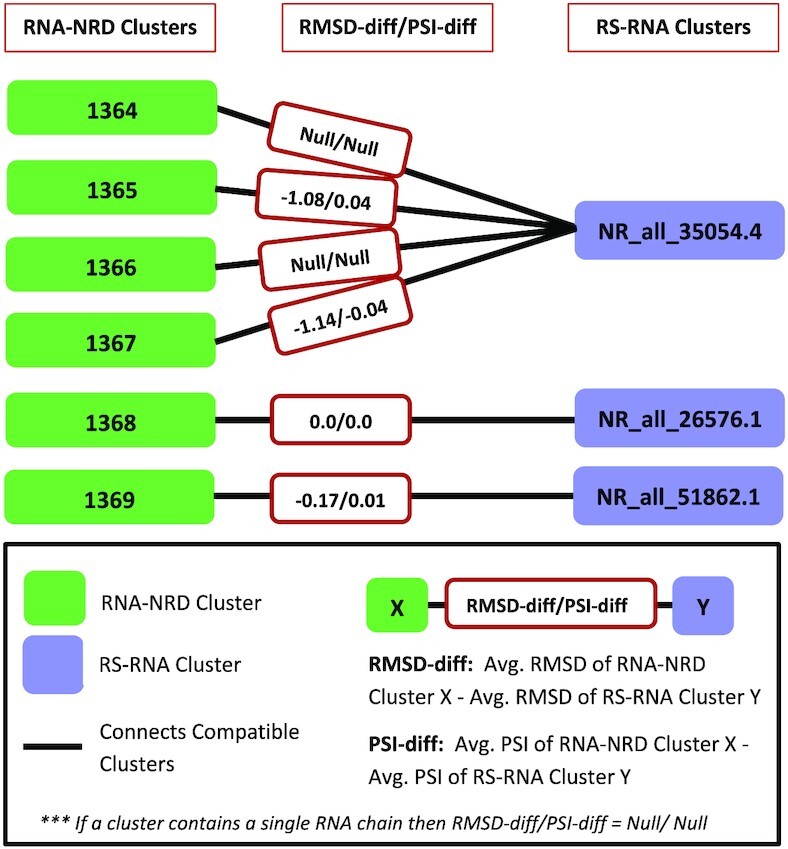
Comparison between compatible clusters of dataset RNA-NRD and RS-RNA demonstrated using an example of organism *Oceanobacillus iheyensis*. The green boxes represent clusters from RNA-NRD dataset while the blue boxes present clusters from RS-RNA dataset. The presence of a solid black line between two clusters means they are compatible. The red-bordered boxes hold the values of RMSD-diff and PSI-diff.

Figure 5 further explains the parameters used in Table 3. From Figure 5, we can see that the organism *Oceanobacillus iheyensis* has six clusters in the RNA-NRD dataset. These six clusters are compatible to three clusters in the RS-RNA dataset. Here, clusters 1364, 1365, 1366, 1367 are compatible with cluster NR_all_35054.4, cluster 1368 is compatible with cluster NR_all_2676.1, and cluster 1369 is compatible with NR_all_51862.1. For this organism, the number of clusters in RNA-NRD having positive (‘+’), negative (‘-’), and zero (‘=’) RMSD-diff is 0, 3, 1 consecutively. Similarly, the number of clusters having positive (‘+’), negative (‘-’) and zero (‘=’) PSI-diff is 2, 1 and 0. The number of single-chain clusters is 2 as cluster 1364 and 1366 contain one chain each. So both their RMSD-diff and PSI-diff values are represented with ‘Null’. The total absolute value of positive RMSD-diff (RMSD +) is 0, negative RMSD-diff (RMSD -) is 1.08 + 1.18 + 0.17 = 2.39, positive PSI-diff (PSI +) is 0.04 + 0.01 = 0.05, and negative PSI-diff (PSI -) is 0.04 for this organism. As the total of ‘RMSD -’ and ‘PSI +’ are higher for the clusters belonging to the RNA-NRD dataset compared to their compatible clusters from the RS-RNA dataset, it can be said that RNA-NRD clusters have better structural similarity compared to the RS-RNA clusters for the organism *Oceanobacillus iheyensis*. As can be seen from Table 3, four organisms have higher absolute value for total ‘- RMSD’ and ‘+ PSI’, meaning for these organisms our dataset RNA-NRD performs better. Three organisms have equal total RMSD and PSI value while RS-RNA dataset performs better for the remaining two organisms. Also, using the RNA-align tool, Table 3 shows that the same four organisms perform better for RNA-NRD and the same three organisms perform equally for both datasets. Overall, our dataset RNA-NRD performs better than RS-RNA based on both RMSD-diff/PSI-diff parameters and RNA-align tool analysis. This shows that the clusters formed in the RNA-NRD dataset are structurally more similar and well-formed compared to the clusters in the RS-RNA dataset.

#### RNAMotifContrast output based comparison of RNA-NRD with RS-RNA

The RNAMotifContrast tool is used to capture the structural variations among the well-known 11 motif families (shown in Table 4) by generating subfamilies within each family based on structural similarities of the motifs. RNAMotifContrast only considers non-redundant RNA chain representatives for collecting motif data as redundant RNA chains can cause data bias while analyzing significant features of RNA structural motifs. So, the input dataset of RNAMotifContrast is curated from a non-redundant dataset. RNAMotifContrast input consists of the locations of 3D motifs in RNA structures. In order to generate these locations, first the RNA instances of well-known motif families are collected which we call annotated RNAs. Then, RNA representatives from the input non-redundant dataset for those annotated RNAs are collected and RNA motifs locations only from these representatives are generated to get rid of overlapping RNA motifs. RNAMotifContrast compares the RNA motifs within each motif family and divides them into motif subfamilies based on structural variations. In an ideal case, subfamilies should not contain multiple RNA motifs that come from RNA chains that are redundant to each other, rather it should contain motifs collected from the representative RNA chain for a set of redundant RNA chains. But if a non-redundant dataset contains redundancy, then the subfamilies generated by RNAMotifContrast will also contain multiple RNA motifs from redundant RNA chains. Based on this understanding, we performed a qualitative comparison of the RS-RNA and the RNA-NRD dataset using the tool RNAMotifContrast to determine which dataset contains less amount of redundancy. Two different sets of subfamily outputs are generated for non-redundant dataset RS-RNA and RNA-NRD using tool RNAMotifContrast. Here, if a subfamily generated by RNAMotifContrast contains multiple RNA motifs from redundant RNA chains, then the subfamily is considered to have redundancy. The generated outputs are analyzed for possible cases of redundancy within the subfamilies. For each subfamily, the sequence and structure of the source RNA chains of the motifs within the same organism are compared against each other using RNAalign tool. Table 4 shows the number of subfamily, redundant subfamily (subfamily containing redundancy), total RNA chain pairs within the same organism, and redundant RNA chain pairs within the same organism for both outputs generated using the RS-RNA and the RNA-NRD datasets. Both the numbers of redundant subfamilies and redundant RNA chain pairs within the same organism are higher in the subfamilies generated from RS-RNA. This means RNA-NRD contains less redundancy compared to RS-RNA. For both the RS-RNA and the RNA-NRD datasets, a list of redundant RNA chain pairs within the subfamilies have been shown in Supplementary Table S1 and S2. To give a better idea, the redundancy comparison of these 11 motif families has been summarized in Table 5. Table 5 shows that using the RNA-NRD dataset, the percentage of non-redundancy is higher for motif family, subfamily, and RNA chain pairs with similar organism. This indicates that our RNA-NRD performs better in removing redundancy and hence generates motif subfamilies with less amount of redundancy.

### RNA-NRD-without-Organism-Division dataset

The first version of the RNA-NRD-without-Organism-Division dataset consists of 9705 RNA chains divided into 2749 clusters. Similar to RNA-NRD, this dataset also contains source organism, macromolecule name, Rfam family name and only contains RNA chains having a length ≥20 nucleotides. The RNA-NRD-without-Organism-Division dataset is shown in Supplementary Table S4.

### Updated version of RNA-NRD dataset

The updated version of the RNA-NRD dataset is generated by merging the unmodified pre-existing clusters from the previous version, modified clusters from the previous version, and the newly generated clusters. This version will contain all the previously existing RNA chains and newly introduced RNA chains in PDB. For this work, an updated version of the RNA-NRD dataset have been generated based on the data collected from PDB on 23 February 2022. This new version is called RNA-NRD version 2. Version 2 of the RNA-NRD dataset is shown in Supplementary Table S5. In version 2 of RNA-NRD, former cluster 1093 and 1098 of version 1 have been removed as they contained PDB entries 6N5R and 6N5L which were later removed from PDB. RNA-NRD version 2 contains a total of 10917 RNA chains which are divided into 3333 clusters. Here, 3006 clusters are added from RNA-NRD version 1 without any modification, 149 clusters are modified as they contain some new RNA chains and 178 new clusters are generated from the new RNA chains which do not have similarities with any existing representatives of version 1. Similarly, an updated version of the RNA-NRD-without-Organism-Division dataset have been generated based on the data collected from PDB on 23 February 2022. This is referred to as RNA-NRD-without-Organism-Division version 2 and contains a total of 10917 RNA chains which are divided into 2881 clusters. Supplementary Table S6 shows version 2 of the RNA-NRD-without-Organism-Division dataset.

### Run time

Using parallel processing we made the pipeline faster. We ran the pipeline on an Ubuntu 20.04.5 system machine with Intel Xeon CPU and 132 GB RAM. It took approximately 45 days to generate the first version of RNA-NRD dataset and an additional 6 days to generate RNA-NRD-without-Organism-Division dataset. The RNA-NRD update pipeline is computationally very fast as it only compares the new RNA chains in PDB. For 50 new RNA chains in PDB, the update pipeline takes around 4-5 hours to generate the new version of non-redundant dataset. So, regular maintenance of this dataset is fast and feasible.

## DISCUSSION

RNA-NRD dataset focuses on both sequence identity and 3D structure similarity for removing redundant RNA structures from PDB database. However, common knowledge is that RNA 3D structure is way more conserved in comparison to RNA sequence. In the analysis done by Capriotti *et al.*, they showed that, with a sequence identity lower than 40%, the core structure of related RNA molecules tend to diverge more significantly (35). But there is no specific sequence-identity cut-off value guaranteeing noticeable 3D structure divergence between two RNA chains. In future, we plan to analyze the relationship between RNA pair sequence identity and 3D structural similarity more closely. Based on that, we plan to propose another version of non-redundant RNA dataset which will focus on structural conservation. In the current version of RNA-NRD, 738 RNA chains having no specific source organism are assigned under the title ‘others’ and analyzed together. In future, we plan to predict the source organisms of such unidentified RNA chains by comparing the representative of their cluster with the representatives belonging to known organism clusters. Another possible improvement on the current dataset will be discarding PDB RNA chains having more than 95% missing coordinate data. Currently, only the RNA-NRD ‘update pipeline’ takes this into consideration.

As research related to RNA functionality and structure analysis is highly dependent on RNA structural data, having an organized non-redundant RNA 3D structure dataset such as RNA-NRD is utterly important. Existing dataset that serves similar purpose are mostly being used in sequence-based or secondary structure based research. But for 3D structure based research, it is important to have more sensitive and accurate dataset. In RNA-NRD, all the RNA chains having similar sequence and 3D structure are put into the same cluster and represented with a single chain. This dataset can be utilized to train machine learning models to predict and model 3D structures with higher accuracy. On top of that, it might be possible to draw a RNA 3D structure-function relation by analyzing the functions of RNA chains within the same cluster identified as redundant. If most of the RNA chains within a cluster tend to perform similar kinds of functionalities, then the function of unfamiliar RNA chains can be predicted based on the clusters they belong to. This will open a new direction in the analysis and prediction of RNA functionality. Apart from that, RNA-NRD can help solve the missing residue coordinate issues in RNA 3D structure databases. As the RNA chains within a redundant cluster are highly structurally similar to one another, the missing coordinates of a closely related RNA chain in a redundant cluster can be predicted based on the structure of representative RNA of that cluster.

Another important utility of RNA-NRD is generating RNA chain families based on their 3D structural and functional similarity. This will help characterize the newly discovered RNA chains by assigning them to a pre-existing RNA family based on features like sequence identity, RMSD, and Alignment Ratio. Research related to RNA evolution using homology-based methods will also be benefited as this dataset tends to cluster the homologous RNA chains together. Research in the field of RNA motif discovery and analysis will be benefited as instead of going through each and every RNA chain, just analyzing the representative RNA chains will give a good initial idea. Thus, RNA-NRD will be very helpful in reducing the computational complexity of further research works done in this field. Overall, the importance of a reliable non-redundant dataset for RNA 3D structures is beyond any doubt. Using different structural alignment tools, quality assessment parameters, and application-based analysis, we show in this paper that compared to the other existing non-redundant 3D structure dataset, RNA-NRD has better formed clusters based on structural similarity and higher percentage of non-redundancy which makes RNA-NRD more reliable as a non-redundant dataset. Thus, RNA-NRD can be highly beneficial to the future endeavors and research in the field of RNA 3D structure.

## DATA AVAILABILITY

RNA-NRD source code is available on Github (https://github.com/ucfcbb/RNA-NRD) and Zenodo (https://doi.org/10.5281/zenodo.7806754). The RNA-NRD datasets and the RNA-NRD-without-Organism-Division datasets are available in the Supplementary Data section. The regular updates of the RNA-NRD dataset are available on the website (http://genome.ucf.edu/RNA-NRD).

## Supplementary Material

lqad040_Supplemental_Files

## References

[1] Al-Hashimi H.M. , Walter N.G. RNA dynamics: it is about time. Curr. Opin. Struct. Biol. 2008; 18:321–329. 18547802 10.1016/j.sbi.2008.04.004 PMC2580758

[2] Mustoe A.M. , Brooks C.L., Al-Hashimi H.M. Hierarchy of RNA functional dynamics. Annu. Rev. Biochem. 2014; 83:441–466. 24606137 10.1146/annurev-biochem-060713-035524 PMC4048628

[3] Berman H.M. , Westbrook J., Feng Z., Gilliland G., Bhat T.N., Weissig H., Shindyalov I.N., Bourne P.E. The protein data bank. Nucleic Acids Res. 2000; 28:235–242. 10592235 10.1093/nar/28.1.235 PMC102472

[4] Burra P.V. , Zhang Y., Godzik A., Stec B. Global distribution of conformational states derived from redundant models in the PDB points to non-uniqueness of the protein structure. Proc. Natl. Acad. Sci. U.S.A. 2009; 106:10505–10510. 19553204 10.1073/pnas.0812152106 PMC2705611

[5] Griep S. , Hobohm U. PDBselect 1992–2009 and PDBfilter-select. Nucleic Acids Res. 2010; 38:D318–D319. 19783827 10.1093/nar/gkp786 PMC2808879

[6] Heringa J. , Sommerfeldt H., Higgins D., Argos P. OBSTRUCT: a program to obtain largest cliques from a protein sequence set according to structural resolution and sequence similarity. Bioinformatics. 1992; 8:599–600. 10.1093/bioinformatics/8.6.599 1468020

[7] Hobohm U. , Scharf M., Schneider R., Sander C. Selection of representative protein data sets. Protein Sci. 1992; 1:409–417. 1304348 10.1002/pro.5560010313 PMC2142204

[8] Lo Conte L. , Ailey B., Hubbard T.J., Brenner S.E., Murzin A.G., Chothia C. SCOP: a structural classification of proteins database. Nucleic Acids Res. 2000; 28:257–259. 10592240 10.1093/nar/28.1.257 PMC102479

[9] Orengo C.A. , Michie A.D., Jones S., Jones D.T., Swindells M.B., Thornton J.M. CATH–a hierarchic classification of protein domain structures. Structure. 1997; 5:1093–1109. 9309224 10.1016/s0969-2126(97)00260-8

[10] He Z. , Zhang C., Xu Y., Zeng S., Zhang J., Xu D. MUFOLD-DB: a processed protein structure database for protein structure prediction and analysis. BMC Genomics. 2014; 15:S2. 10.1186/1471-2164-15-S11-S2 PMC4304177 25559128

[11] Miao Z. , Westhof E. RNA structure: advances and assessment of 3D structure prediction. Annu. Rev. Biophys. 2017; 46:483–503. 28375730 10.1146/annurev-biophys-070816-034125

[12] Zhang Y. , Wang J., Xiao Y. 3dRNA: 3D structure prediction from linear to circular RNAs. J. Mol. Biol. 2022; 434:167452. 35662453 10.1016/j.jmb.2022.167452

[13] Dawson W.K. , Bujnicki J.M. Computational modeling of RNA 3D structures and interactions. Curr. Opin. Struct. Biol. 2016; 37:22–28. 26689764 10.1016/j.sbi.2015.11.007

[14] Islam S. , Rahaman M.M., Zhang S. RNAMotifContrast: a method to discover and visualize RNA structural motif subfamilies. Nucleic Acids Res. 2021; 49:e61–e61. 33693841 10.1093/nar/gkab131 PMC8216276

[15] Klosterman P.S. , Tamura M., Holbrook S.R., Brenner S.E. SCOR: a structural classification of RNA database. Nucleic Acids Res. 2002; 30:392–394. 11752346 10.1093/nar/30.1.392 PMC99131

[16] Murthy V.L. , Rose G.D. RNABase: an annotated database of RNA structures. Nucleic Acids Res. 2003; 31:502–504. 12520063 10.1093/nar/gkg012 PMC165459

[17] Tamura M. , Hendrix D.K., Klosterman P.S., Schimmelman N.R., Brenner S.E., Holbrook S.R. SCOR: Structural Classification of RNA, version 2.0. Nucleic Acids Res. 2004; 32:D182–D184. 14681389 10.1093/nar/gkh080 PMC308814

[18] Sarver M. , Zirbel C.L., Stombaugh J., Mokdad A., Leontis N.B. FR3D: finding local and composite recurrent structural motifs in RNA 3D structures. J. Math. Biol. 2008; 56:215–252. 17694311 10.1007/s00285-007-0110-x PMC2837920

[19] Yang L. , Liu Y., Hu X., Wang P., Li X., Wu J. Graph-based analysis of RNA secondary structure similarity comparison. Complexity. 2021; 2021:8841822.

[20] Lu X.-J. , Olson W.K. 3DNA: a software package for the analysis, rebuilding and visualization of three-dimensional nucleic acid structures. Nucleic Acids Res. 2003; 31:5108–5121. 12930962 10.1093/nar/gkg680 PMC212791

[21] Das J. , Mukherjee S., Mitra A., Bhattacharyya D. Non-canonical base pairs and higher order structures in nucleic acids: crystal structure database analysis. J. Biomol. Struct. Dyn. 2006; 24:149–161. 16928138 10.1080/07391102.2006.10507108

[22] Roy A. , Panigrahi S., Bhattacharyya M., Bhattacharyya D. Structure, stability, and dynamics of canonical and noncanonical base pairs: quantum chemical studies. J. Phys. Chem. B. 2008; 112:3786–3796. 18318519 10.1021/jp076921e

[23] Abraham M. , Dror O., Nussinov R., Wolfson H.J. Analysis and classification of RNA tertiary structures. RNA. 2008; 14:2274–2289. 18824509 10.1261/rna.853208 PMC2578864

[24] Ray S.S. , Halder S., Kaypee S., Bhattacharyya D. HD-RNAS: an automated hierarchical database of RNA structures. Front. Genet. 2012; 3:59. 22529851 10.3389/fgene.2012.00059 PMC3329738

[25] Leontis N.B. , Zirbel C.L. Nonredundant 3D structure datasets for RNA knowledge extraction and benchmarking. RNA 3D Structure Analysis and Prediction. 2012; Springer 281–298.

[26] Schrödinger LLC 2015; The PyMOL Molecular Graphics System, Version 1.8.

[27] Griffiths-Jones S. , Bateman A., Marshall M., Khanna A., Eddy S.R. Rfam: an RNA family database. Nucleic Acids Res. 2003; 31:439–441. 12520045 10.1093/nar/gkg006 PMC165453

[28] Gardner P.P. , Daub J., Tate J.G., Nawrocki E.P., Kolbe D.L., Lindgreen S., Wilkinson A.C., Finn R.D., Griffiths-Jones S., Eddy S.R. et al. Rfam: updates to the RNA families database. Nucleic Acids Res. 2009; 37:D136–D140. 18953034 10.1093/nar/gkn766 PMC2686503

[29] Burge S.W. , Daub J., Eberhardt R., Tate J., Barquist L., Nawrocki E.P., Eddy S.R., Gardner P.P., Bateman A. Rfam 11.0: 10 years of RNA families. Nucleic Acids Res. 2013; 41:D226–D232. 23125362 10.1093/nar/gks1005 PMC3531072

[30] Nawrocki E.P. , Burge S.W., Bateman A., Daub J., Eberhardt R.Y., Eddy S.R., Floden E.W., Gardner P.P., Jones T.A., Tate J. et al. Rfam 12.0: updates to the RNA families database. Nucleic Acids Res. 2015; 43:D130–D137. 25392425 10.1093/nar/gku1063 PMC4383904

[31] Kalvari I. , Argasinska J., Quinones-Olvera N., Nawrocki E.P., Rivas E., Eddy S.R., Bateman A., Finn R.D., Petrov A.I. Rfam 13.0: shifting to a genome-centric resource for non-coding RNA families. Nucleic Acids Res. 2018; 46:D335–D342. 29112718 10.1093/nar/gkx1038 PMC5753348

[32] Kalvari I. , Nawrocki E.P., Ontiveros-Palacios N., Argasinska J., Lamkiewicz K., Marz M., Griffiths-Jones S., Toffano-Nioche C., Gautheret D., Weinberg Z. et al. Rfam 14: expanded coverage of metagenomic, viral and microRNA families. Nucleic Acids Res. 2021; 49:D192–D200. 33211869 10.1093/nar/gkaa1047 PMC7779021

[33] Leinonen R. , Akhtar R., Birney E., Bower L., Cerdeno-Tárraga A., Cheng Y., Cleland I., Faruque N., Goodgame N., Gibson R. et al. The European nucleotide archive. Nucleic Acids Res. 2010; 39:D28–D31. 20972220 10.1093/nar/gkq967 PMC3013801

[34] Needleman S.B. , Wunsch C.D. A general method applicable to the search for similarities in the amino acid sequence of two proteins. J. Mol. Biol. 1970; 48:443–453. 5420325 10.1016/0022-2836(70)90057-4

[35] Capriotti E. , Marti-Renom M.A. Quantifying the relationship between sequence and three-dimensional structure conservation in RNA. BMC Bioinformatics. 2010; 11:322. 20550657 10.1186/1471-2105-11-322 PMC2904352

[36] Laederach A. Informatics challenges in structured RNA. Brief. Bioinform. 2007; 8:294–303. 17611237 10.1093/bib/bbm026 PMC2629073

[37] Ge P. , Zhang S. STAR3D: a stack-based RNA 3D structural alignment tool. Nucleic Acids Res. 2015; 43:e137. 26184875 10.1093/nar/gkv697 PMC4787758

[38] Lu X.J. , Bussemaker H.J., Olson W.K. DSSR: an integrated software tool for dissecting the spatial structure of RNA. Nucleic Acids Res. 2015; 43:e142. 26184874 10.1093/nar/gkv716 PMC4666379

[39] Smyth M. , Martin J. x Ray crystallography. Mol. pathol. 2000; 53:8–14. 10884915 10.1136/mp.53.1.8 PMC1186895

[40] Domagalski M.J. , Zheng H., Zimmerman M.D., Dauter Z., Wlodawer A., Minor W. The quality and validation of structures from structural genomics. Structural Genomics. 2014; Springer 297–314. 10.1007/978-1-62703-691-7_21 PMC4081469 24203341

[41] Gong S. , Zhang C., Zhang Y. RNA-align: quick and accurate alignment of RNA 3D structures based on size-independent TM-scoreRNA. Bioinformatics. 2019; 35:4459–4461. 31161212 10.1093/bioinformatics/btz282 PMC6821192

