# Supplementary material for: RNA-NRD: a non-redundant RNA structural dataset for benchmarking and functional analysis
Source: NAR Genom Bioinform. 2023 Apr 26;5(2):lqad040. doi: 10.1093/nargab/lqad040 (PMC10132383; doi:10.1093/nargab/lqad040)
Supplement: lqad040_Supplemental_Files [file lqad040_supplemental_files.zip › RNA-NRD_Supplementary_Data.pdf]

Supplementary Data for “RNA-NRD: a Non-redundant RNA Structural Dataset for Benchmarking and Functional Analysis”

Nabila Shahnaz Khan, Md Mahfuzur Rahaman, Shahidul Islam and Shaojie Zhang *

Table S1: Redundant RNA chain pairs within motif subfamilies generated by RNAmotifContrast using RS-RNA dataset

Family	Subfamily	Organism	RNA chain pairs	RNA loops	Sequence Identity %	RMSD
C-loop	Sub1	Other	4JRC_B:4MGN_A	4JRC_B:29-33_73-75, 4MGN_A:29-33_73-75	92.9	1.58
E-loop	Sub1	Other	3IWN_A:3MXH_R	3IWN_A:12-16_31-35, 3MXH_R:22-26_41-45	91.6	1.48
		Other	3DHS_A:2A64_A	3DHS_A:284-288_297-301, 2A64_A:284-288_297-301, 2A64_A:347-351_378-382	96.7	1.77
Kink-turn	Sub1	*Thermus thermophilus*	4Y4O_2A:3U4M_B	4Y4O_2A:1208-1215_1234-1238, 4Y4O_2A:81-85_97-105, 3U4M_B:2129-2136_2155-2159	93.7	2.46
		Caldanaerobacter subterraneus	2YGH_A:5FJC_A	2YGH_A:17-21_31-38, 5FJC_A:17-21_31-38	93.5	0.71
		Caldanaerobacter subterraneus	2YGH_A:3V7E_C	2YGH_A:17-21_31-38, 3V7E_C:17-21_31-38	91.2	2.41
		Pyrococcus furiosus	3NVI_F:3NMU_E	3NVI_F:5-8_15-21, 3NMU_E:14-17_24-30	100	0.61
		Caldanaerobacter subterraneus	5FJC_A:3V7E_C	5FJC_A:17-21_31-38, 3V7E_C:17-21_31-38	87.8	2.5
L1-complex	Sub1	Other	2HW8_B:1U63_D	2HW8_B:10-13_22-29, 1U63_D:10-13_33-40	83.3	1.8
Sarcin-ricin	Sub1	Other	3D0U_A:3DIL_A	3D0U_A:20-27_60-66, 3DIL_A:22-30_63-70	91.3	1.85
Tandem-shear	Sub1	Other	3D0U_A:3DIL_A	3D0U_A:16-19_67-70, 3DIL_A:19-22_70-73	91.3	1.85

Table S2: Redundant RNA chain pairs within motif subfamilies generated by RNAmotifContrast using RNA-NRD dataset

Family	Subfamily	Organism	RNA chain pairs	RNA loops	Sequence Identity %	RMSD
C-loop	Sub1	Other	4JRC_B:4MGN_A	4JRC_B:29-33_73-75, 4MGN_A:29-33_73-75	92.9	1.58
	Sub4	*Escherichia coli*	6WNV_4:4V71_BB	6WNV_4:1974-1977_2021-2022, 4V71_BB:30-31_51-54	97.7	2.07
E-loop	Sub1	Other	3DHS_A:2A64_A	3DHS_A:284-288_297-301, 2A64_A:284-288_297-301, 2A64_A:347-351_378-382	96.7	1.77
Kink-turn	Sub1	*Thermus thermophilus*	4V8E_AA:5NPM_B	4V8E_AA:1208-1215_1234-1238, 5NPM_B:25-32_51-55	92.5	2.16
		Caldanaerobacter subterraneus	5FK5_A:3V7E_D	5FK5_A:17-21_31-38, 3V7E_D:217-221_231-238	89.1	2.12
		Pyrococcus furiosus	3NVI_E:3NMU_E	3NVI_E:5-8_15-21, 3NMU_E:14-17_24-30	100	0.60
L1-complex	Sub1	Other	2HW8_B:1U63_B	2HW8_B:10-13_22-29, 1U63_B:10-13_33-40	85.7	1.61

*To whom correspondence should be addressed. Tel: +1 407 823 6095; Fax: +1 407 823 5835; Email: shzhang@cs.ucf.edu
